# Mental health in children with disabilities and their families: red flags, services' impact, facilitators, barriers, and proposed solutions

**DOI:** 10.3389/fresc.2024.1347412

**Published:** 2024-02-12

**Authors:** Kayla Heslon, Jessica Helena Hanson, Tatiana Ogourtsova

**Affiliations:** ^1^ Integrated Center of Health and Social Services of Laval, The Research Center of the Jewish Rehabilitation Hospital, Laval, QC, Canada; ^2^ Faculty of Arts and Sciences, McGill University, Montreal, QC, Canada; ^3^ Faculty of Medicine and Health Sciences, School of Physical and Occupational Therapy, McGill University, Montreal, QC, Canada; ^4^ Centre for Interdisciplinary Research in Rehabilitation of Greater Montreal, Montreal, QC, Canada

**Keywords:** neurodevelopmental disabilities, mental health, wellbeing, rehabilitation, qualitative

## Abstract

**Background:**

Children and youth with neurodevelopmental disabilities (NDDs) and their caregivers are at a high risk of experiencing mental health challenges, that in turn can significantly affect their functioning, productivity, and quality of life. In this already vulnerable population, mental health difficulties are now more frequently reported and pronounced secondary to the isolation and uncertainties experienced during the pandemic. Our previous work has shown important mental health services' gaps for children/youth with NDDs and their families, highlighting the need to optimize and tailor existing practices.

**Objective:**

To explore mental health services' barriers, facilitators, impact, and solutions from the perspectives of HCPs and CGs, and to describe common precursors to mental health challenges in children with NDDs from the perspectives of these two groups.

**Methods:**

In a triangulation mixed-method study design embedding quantitative and qualitative approaches, participants completed a survey and a semi-structured interview. Descriptive statistics and a hybrid inductive/deductive thematic approach were used for data analysis.

**Results:**

Over 700 utterances were analyzed (247 from caregivers [*n* = 10], 531 from clinicians [*n* = 16]) and included 143 and 173 statements related to the precursors and barriers/facilitators, respectively. Common precursors to mental health challenges (*n* = 7 categories) were identified and included reported feelings/perception of self, behavioral and physical manifestations, emotional dysregulation, and school-related factors, among others. Clinicians reported a widespread need for pediatric, family-centered mental health services and conveyed lacking mental health resources/training to meet the demand. Caregivers indicated being only moderately satisfied when care was received. Salient facilitators identified by clinicians were having an interdisciplinary team and caregiver's engagement in the therapeutic processes. Participants recommended improvements to increase accessibility to mediate the existing discrepancy between the emergence of precursors and care received; that services must target a broader population and be more comprehensive (e.g., family-centered care, addressing high-risk transition periods); and training/toolkits to support clinicians’ evidence-based practice.

**Conclusion:**

Our findings emphasize the necessity of a systematic and standardized approach to mental health services for children with NDDs and their families. Enhancing caregiver support, addressing barriers, and adopting a proactive, family-centered approach are crucial for improving accessibility and quality. These proposed solutions provide valuable insights for shaping policies and practices in pediatric mental health services.

## 1 Introduction

Neurodevelopmental disabilities (NDDs, e.g., autism spectrum disorder, cerebral palsy), characterized by cognitive, behavioral, language and/or motor abilities impairments, significantly affect children's daily performance, productivity, and participation (1, 2). In addition, children with NDDs are also more likely to present with co-existing mental health issues (e.g., anxiety, depression) compared to their typically developing peers (3), where a higher incidence (30%–50% vs. 8%–18%) of mental health disorders is reported (4). For instance, toddlers with developmental delays experience more emotional and behavioral difficulties (5), where by age of five years old, children with developmental delays demonstrate increased rates of psychological disorders compared to typically developing peers (6). In fact, approximately 70% of mental health challenges emerge in childhood or adolescence, with persistent issues into adulthood (7) that in turn, result in long-lasting and significant effects on the individual's health and social factors (8). Additionally, caregivers of children with NDDs are at a high risk of experiencing substantial mental health issues as they need to adjust and adapt to their child's frequently changing needs (9, 10). Mothers, in particular, show heightened mental health concerns (11), with reports of anxiety about their child's development, social stigma, and discrimination (12–14). Overall, these mental health concerns in children and their caregivers are known to adversely affect their performance, productivity, and quality of life (15). Furthermore, the significant socioeconomic repercussions of pediatric mental health disorders underscore the importance of the issue, with an estimated lifetime cost amounting to $2.1 trillion USD (16).

The COVID-19 pandemic has exacerbated mental health concerns for children with NDDs and caregivers alike (17, 18), heightening the need for services (19). Nevertheless, the latest scoping review on the organization and the delivery of mental health services for youth with physical NDDs such as cerebral palsy highlighted a lack of models of care that are fully integrated into pediatric rehabilitation settings (20), with only one example from Canada (21). This further supports existing evidence that pediatric mental health care is primarily distinct from the biomedical care, particularly in pediatric dual diagnosis (22). This raises significant concern since continuous and well-coordinated rehabilitation services are crucial for children with NDDs, particularly during transition periods (e.g., school entry, adolescence) (23), where many mental health disorders emerge for the first time (24).

Recently, our team highlighted gaps in existing in-person and telehealth/online mental health services available locally (Montreal/Laval/Laurentians, Quebec, Canada) (25) and beyond (26, 27) for children with NDDs and their families, emphasizing the necessity for enhancing the design and delivery of existing services. Our environmental scan showed that 47% of in-person local mental health pediatric programs had specific admission criteria (e.g., diagnosis, age limit), 67% required a formal referral (e.g., from a primary care physician), and nearly 40% had a waitlist to access services provided the child fits the admission criteria and has a referral (25). Furthermore, our systematic review identified that none of the existing telerehabilitation programs were targeting to improve mental health issues in children with physical NDDs (e.g., cerebral palsy) (27). Likewise, a 2022 scoping review focusing on mental health challenges in youths with childhood-onset physical NDDs evidenced that only a small number of individuals with mental health concerns accessed services and identified numerous mental health care needs that remain unaddressed (28).

The concept of preventative vs. reactive care is relevant to the mental health of children/youth with NDDs and their families. It has been suggested that to promote improved psychosocial adjustment for individuals with NDDs such as intellectual disability, it is crucial to incorporate mental health prevention and early detection into healthcare policies (29). Targeted prevention (i.e., for those with clearly recognized risk factors such as an NDD) heavily relies on individuals in close proximity and contact with the child (e.g., caregiver, educator, teacher, community workers, primary health providers) (30). However, there is evidence that despite the high prevalence of mental health concerns, the identification of these issues is often missed by professional and family-caregivers, as well as paraprofessionals (31, 32). It is therefore relevant to explore potential warning signs of mental health challenges in children with NDDs and implement effective knowledge mobilization strategies to increase awareness among individuals in close proximity to the child and to promote preventative care.

On a larger scope, it becomes evident that there is a “broken bridge” between the needs (i.e., widespread occurrence of mental health issues in children with NDDs and their families) and the provision of services (including availability, accessibility, and comprehensiveness), where the “fall” can result in long-term devastating impact for this already vulnerable population. While environmental scans, reviews, and a framework of mental health services (20, 21, 25, 28) have been conducted and proposed in efforts to optimize services delivery and outcomes, the perspectives of key stakeholders are yet to be explored. These include pediatric healthcare practitioners (HCPs) working in rehabilitation settings with children with NDDs and their families, as well as the caregivers (CGs) of children with NDDs. Understanding their perspectives could serve as a valuable tool to advocate for the needed changes in preventative and therapeutic mental health care for children and youth with NDDs and their families. As the next step in improving mental health care for this population, our objectives were to explore mental health services' barriers, facilitators, impact, and solutions from the perspectives of HCPs and CGs. Additionally, as a stepping stone to enhancing awareness and promoting targeted prevention, we aimed to describe common mental health triggers (“red flags”/precursors) in children with NDDs from the perspectives of these two groups.

## 2 Methods

### 2.1 Study design

A triangulation mixed-method study design (33) was employed and included quantitative (questionnaire) and qualitative (semi-structured interviews) approaches.

### 2.2 Recruitment sites & participants

HCPs and CGs were recruited through four partnering sites. These were outpatient pediatric rehabilitation settings, providing care to children and youth with NDDs and their families in Greater Montreal/Laval/Laurentians areas, Quebec, Canada. These settings serve children and youth in three age categories (0–6, 7–15, and 16–21 years of age) under one umbrella of services for intellectual/physical/autism spectrum-related disabilities according to the following three service trajectories: (1) Developmental; (2) Language and Communication; (3) Interactions and Social Communication. Different HCPs are part of the multidisciplinary teams, including physical (PT), occupational therapists (OT), speech language pathologists (SLPs), social workers/case managers (SW), psychologists and neuropsychologists, special educators, nutritionists, recreational and behavioral therapists. The location was selected to understand the contextual experiences within a provincial healthcare jurisdiction.

HCPs were included if they were licensed professionals working in pediatric rehabilitation for at least six months before recruitment and addressed/considered patients’ mental health in their practice (e.g., screening, assessment, referral, intervention, planning). The disciplines of the HCPs recruited are representative of the multidisciplinary care teams intervening with the target population of children with NDDs and their families (25). CGs were included if they had children/youth with NDDs and had experience with services pertaining to their child's mental health in the year before recruitment (e.g., attempting to access mental health assessment for their child). It has been recommended that for qualitative interview studies, a sample size between 9 and 17 participants is necessary to reach data saturation (34). Therefore, a minimum sample size of 9 participants per group or until data saturation, for a total of 18 participants minimum, was deemed sufficient for the qualitative analysis and scale of this study. The study was approved by a recognized ethics review board (REB CRIR #MP-50-2023-1681), and all participants reviewed and signed the consent form.

### 2.3 Study procedures

The procedure included three main steps (study materials available in Supplementary File S1). For both study groups, following the completion of a demographic survey, we used an in-house-developed 4-point Likert Scale (ranging from *Not at all/Never* to *Very much/All the time*) to explore participants' awareness about and accessibility to mental health services, and satisfaction with these services. The survey was reviewed by a clinical expert (pediatric OT) for suitability and readability prior to deployment. Lastly, we conducted semi-structured interviews using a pre-designed interview guide to explore the provided or received services, red flags to mental health issues, barriers and facilitators to mental health services and management, impact of services, and optimizing solutions. The interview guide and survey for CGs were co-developed and validated with four parent-partners (caregivers of children with NDDs) to ensure high suitability and user-friendliness.

### 2.4 Analysis

The quantitative data analysis used descriptive statistics (proportions/counts, means, standard deviations) via SPSS and Microsoft Excel. Participants' selections on the Likert Scale, along with their demographic variables, were entered for analysis by the first author (KH) and verified for accuracy by senior author (TO).

Semi-structured interviews were transcribed verbatim and imported into the NVivo software (QRS International 12) for analysis by the first author (KH). Identifiers were referred to all participants, and names were removed to ensure the anonymity of the HCP and CG participants and their children. Citations originally in French have been translated and reviewed by two bilingual authors (KH, TO). A hybrid inductive/deductive thematic approach was used (35) alongside a reflexive thematic analysis process (36). To begin, two authors (TO, KH) created the initial themes inductively (based on the interview questions). Following, transcripts were reviewed and analyzed for patterns to determine the emerging salient themes and subthemes by first author (KH). Subsequently, for each transcript, utterances were matched into existing themes and subthemes. Additional theme/subtheme nodes were created when participants' statements did not fit into existing categories. Data verification took place once all the interview transcripts were coded. This entailed examining each theme and subtheme and combining them in cases of overlap. This was performed by the first author (KH). To validate the thematic analysis process and for ambiguous statements, discussions took place with the three authors (KH, JH, and TO). After this iterative process, a final extensive verification/validation procedure was conducted, where each coded statement was verified one by one for its fit in the theme and subtheme by two authors (JH, TO). Discrepancies were resolved through discussion. Qualitative citations were chosen across the participants to include different experiences and backgrounds (whether professional or situational) to increase the reliability and transparency of the data.

To compare and contrast quantitative and qualitative findings, a narrative approach was used, where possible.

## 3 Results

Sixteen (*n* = 16) HCPs and ten CGs (*n* = 10) were recruited between October 2022 and March 2023. Their demographic characteristics are displayed in Table 1 and Table 2. HCPs were all female of various health disciplines, with OTs representing more than half of the sample, aged 40.3 ± 7.7 years old on average, with 12.9 ± 7.7 years of experience. CGs were mostly mothers (80%), aged 45.7 ± 8.0 years old on average, and had children with various NDDs, where autism spectrum disorder represented 38.5% of the sample.

**Table 1:** Demographic variables of study participants.

Groups Interviewed			
Total (*n*)	HCP, *n* = 16	CG, *n* = 10	Children, *n* = 13
Average age (years, mean ± SD)			
	40.3 ± 7.7	45.7 ± 8.0	11.6 ± 3.9
Gender, *n* (%)			
* Female*	16 (100)	8 (80)	8 (61.5)
* Male*	0 (0)	2 (20)	5 (38.5)
Highest educational attainment, *n* (%)			
* Bachelors*	5 (31.3)	5 (50)	–
* Masters*	10 (62.5)	4 (40)	–
* Doctorate*	1 (6.3)	–	–
* Other*	–	1 (10)	–
Years of clinical experience in pediatrics, *n* (%)			
* 5 years and under*	2 (12.5)		
* 6 to 10 years*	5 (31.3)		
* 11 to 20 years*	6 (37.5)		
* 21 years and above*	3 (18.7)		
* Average (mean ± SD* **)**	12.9 ± 7.7		

HCP, health care provider; CG, caregiver; SD, standard deviation.

**Table 2:** Characteristics of study participants.

HCPs (*n* = 16)	
Clinical population, *n* (%)	
General pediatrics	7 (36.8)
Language/communication disorders	4 (21.1)
DCD	3 (15.8)
Motor deficiencies	2 (10.5)
Cerebral palsy	1 (5.3)
Hearing/vision impairments	2 (10.5)
Occupation, *n* (%)	
Occupational therapist	9 (56.3)
Social worker	2 (12.5)
Dietician	1 (6.3)
Psychoeducator	1 (6.3)
Neuropsychologist	1 (6.3)
Speech Therapist	1 (6.3)
Physiotherapist	1 (6.3)
CGs (*n* = 10)	
Occupation, *n* (%)	
Teacher	2 (20)
Project director	1 (10)
Marketing researcher	1 (10)
Consultant	1 (10)
Project manager	1 (10)
Community legal worker	1 (10)
Psychoeducator and clinical coordinator	1 (10)
Other	2 (20)
Children of CGs (*n* = 13)	
Diagnosis of child, *n* (%)	
ASD	5 (38.5)
Language/communication disorders	1 (7.7)
DCD	1 (7.7)
Familial dysautonomia	1 (7.7)
Intellectual disability	1 (7.7)
ADHD	1 (7.7)
Duchenne muscular dystrophy	1 (7.7)

HCP, health care practitioner; CG, caregiver; ASD, autism spectrum disorder; DCD, developmental coordination disorder; ADHD, attention-deficit/hyperactivity disorder.

### 3.1 Survey results

Table 3A outlines the response frequencies related to the awareness and accessibility of mental healthcare services for children with NDDs. Most of the CGs (90%) expressed that their children were “*Often*” on the waitlist for mental health services. Positive and negative experiences with health care services related to their child's mental health were evenly distributed and ranged from “*Never*” to “*All the time*”. Over the past year, most caregivers (80%–100%) reported that the admission criteria (e.g., location, diagnosis) have not impeded services accessibility. While 90% of CGs reported being actively involved in their child's mental health management, 50% of CGs specified being only “*Sometimes*” supported by the HCPs from whom they received care (Table 3B). Once services were accessed, 70% of CGs were “*Moderately*” to “*Very much*” satisfied with HCP communication and time spent understanding and assessing the mental health care needs of the child.

**Table 3A:** Response frequency of CGs: awareness and accessibility of mental health-care services for their child with NDD.

	Not at all *Never* *n* (%)	Slightly *Sometimes* *n* (%)	Moderately often *n* (%)	Very much *All the time* *n* (%)
Over the past year, mental health services for my child have been easily accessible (e.g., location, availability, cost, admission criteria).	2 (20)	4 (40)	3 (30)	1 (10)
Over the past year, mental health care services were declined for my child because they did not meet the admission criteria.	3 (60)	0 (0)	1 (20)	1 (20)
Over the past year, mental health care services were declined for my child because of the location.	4 (100)	0 (0)	0 (0)	0 (0)
Over the past year, I have had to refuse mental health care services because of associated costs (e.g., consulting in a private clinic).	4 (80)	0 (0)	0 (0)	1 (20)
Presently, or in the past, my child has been on a waiting list for mental health services.	1 (10)	0 (0)	4 (40)	5 (50)
Do you feel that you were adequately informed and aware of the mental health services offered for your child?	4 (40)	2 (20)	2 (20)	2 (20)
I had positive/good experiences/helpful interactions with health care services related to my child's mental health.	1 (10)	3 (30)	3 (30)	3 (30)
I had negative/bad experiences/not helpful interactions with health care services related to my child's mental health.	3 (30)	4 (40)	0 (0)	3 (30)

Response frequency (%)	Color-code
0 to <25	
25 to <50	
50 to <75	
75 to 100	

**Table 3B:** Response frequency of CGs: satisfaction with the mental health services offered by HCPs.

	Not at all *Never* *n* (%)	Slightly *Sometimes* *n* (%)	Moderately often *n* (%)	Very much *All the time* *n* (%)
I feel my child's mental health has been generally supported by HCPs.	1 (10)	5 (50)	3 (30)	1 (10)
As a caregiver, I feel generally supported by HCPs in relation to my child's mental health.	2 (20)	3 (30)	4 (40)	1 (10)
Overall, I am satisfied with the mental health services my child has received.	2 (20)	3 (30)	4 (40)	1 (10)
Overall, I am satisfied with the interactions/communication I had with HCPs in the past year about my child's mental health.	2 (20)	2 (20)	3 (30)	3 (30)
I have felt HCPs spent time understanding the situation and addressing my child's mental health needs.	1 (10)	2 (20)	4 (40)	3 (30)
I have felt informed about my child's condition during my child's mental health management support.	2 (20)	4 (40)	1 (10)	3 (30)
I have been actively engaged in my child's mental health management (e.g., being present at evaluation, involved in the choice of treatment options, applying intervention strategies with my child).	1 (10)	0 (0)	0 (0)	9 (90)
The mental health services received have had a positive impact on my child.	1 (10)	3 (30)	4 (40)	2 (20)
The mental health services received have had a positive impact on me as a CG.	3 (30)	3 (30)	3 (30)	1 (10)

Response frequency (%)	Color-code
0 to <25	
25 to <50	
50 to <75	
75 to 100	

Half of participating HCPs reported that children with a range of NDDs would “*Often*” benefit from mental health services, including evaluation, treatment, and follow-up (Table 4A). A higher need for services for children with learning disabilities, speech disabilities, attention-deficit and hyperactivity disorders (ADHD), and developmental coordination disorder (DCD) was conveyed. Moreover, 50% of HCPs reported that caregivers would “*Often*” benefit from mental health services for themselves as part of family-centered care. On average, over 60% of them “*Sometimes*” proceeded to refer to more targeted mental health programs. Importantly, 50% reported feeling only “*Slightly*” prepared to address pediatric mental health challenges and many (87.5%) conveyed being not adequately informed about referral possibilities (Table 4B).

**Table 4A:** Response frequencies of HCPs who over the past year have had pediatric patients with the following condition(s) who would benefit from mental health services (e.g., evaluation, treatment, follow-up).

	Not at all *Never* *n* (%)	Slightly *Sometimes* *n* (%)	Moderately often *n* (%)	Very much *All the time* *n* (%)
Cerebral palsy	5 (35.7)	3 (21.4)	3 (21.4)	3 (21.4)
ASD	3 (21.4)	7 (50)	2 (14.2)	3 (21.4)
ADHD	0 (0)	3 (18.7)	7 (43.7)	6 (37.5%)
DCD	0 (0)	2 (16.6)	4 (33.3)	6 (50)
Learning disability	0 (0)	0 (0)	9 (81.8)	2 (18.8)
Intellectual disability	2 (16.6)	6 (50)	3 (25)	1 (8.3)
Global developmental delay	2 (14.2)	5 (35.7)	5 (35.7)	2 (14.2)
Speech disability	0 (0)	1 (8.3)	7 (58.3)	4 (33.3)
Other	0 (0)	1 (12.5)	3 (37.5)	4 (50)
ASD, autism spectrum disorder; DCD, developmental coordination disorder; ADHD, attention-deficit/hyperactivity disorder.				

Response frequency (%)	Color-code
0 to <25	
25 to <50	
50 to <75	
75 to 100	

**Table 4B:** Response frequencies of HCPs regarding experience with mental health care management.

	Not at all *Never* *n* (%)	Slightly *Sometimes* *n* (%)	Moderately often *n* (%)	Very much *All the time* *n* (%)
Over the past year, I have had parents/caregivers of pediatric patients who would benefit from mental health services as part of family-centered care.	0 (0)	4 (25)	8 (50)	4 (25)
Over the past year, I have had to refer patients and/or their caregivers to more targeted mental health program(s).	0 (0)	10 (62.5)	4 (25)	2 (12.5)
I am adequately prepared to conduct pediatric mental health assessments.	2 (18.1)	4 (36.3)	2 (18.1)	1 (9.0)
I am adequately prepared to treat pediatric mental health challenges.	2 (20)	6 (60)	2 (20)	0 (0)
I am adequately prepared to manage parental mental health issues (e.g., parents’ stress, depression, anxiety, mental breakdown).	3 (25)	5 (41.6)	3 (25)	1 (8.3)
I feel well informed about referral possibilities for targeted mental health issues (e.g., acute crises, group therapy).	2 (12.5)	8 (50)	3 (18.7)	3 (18.7)
I feel supported by my management in addressing mental health challenges in my pediatric clientele.	4 (25)	4 (25)	5 (31.2)	3 (18.7)
I feel well informed about existing mental health care services/resources that are offered within my clinical setting and partnering sites.	1 (6.2)	6 (37.5)	7 (43.7)	2 (12.5)
I feel well informed about existing mental health care services/resources that are offered outside of my clinical setting and partnering sites (e.g., another jurisdiction, online services).	4 (25)	10 (62.5)	1 (6.2)	1 (6.2)

Response frequency (%)	Color-code
0 to <25	
25 to <50	
50 to <75	
75 to 100	

### 3.2 Semi-structured interview results

Through the semi-structured interviews, the following main themes emerged: (1) Red flags/precursors to mental health issues in children with NDDs; (2) Mental health care provided by HCPs (3) Barriers and facilitators to pediatric mental health services; (4) Positive and negative experiences of CGs within the pediatric health care systems; and (5) Solutions to improve mental health services.

#### 3.2.1 THEME: red flags/precursors to mental health issues in children with NDD

One hundred and forty-three statements from both study groups related to red flags or precursors to mental health issues in children with NDDs were coded (Table 5). Seven (*n* = 7) categories were identified. In descending order, from most to least reported, these included (1) Reported feelings and perception of self, with self-esteem/negative self talk, depression and anxiety symptoms as most common; (2) Factors related to school (e.g., isolation and struggles with academic performance); (3) Behavioural manifestations (e.g., poorer sleep patterns, food refusal); (4) Physical manifestations (e.g., agitation or physical aggression); (5) Emotional dysregulation (e.g., tantrums); (6) Factors related to family dynamics (e.g., parental distress, financial strain), and (7) Mental health crises (e.g., panic attack). According to CGs, some precursors to mental health challenges have been flagged by other individuals surrounding the child, for example, grandparents, teachers, and therapists. However, they report being mostly the one who first detect their child's precursor(s).

**Table 5:** Mental health red flags from the perspectives of CGs and HCPs.

Red Flags					
Theme *n* = number of utterances % of Theme	Subtheme	Description	CGs’ utterances *n* (%)	HCPs’ utterances *n* (%)	Total utterances *n* (%)
*Reported feelings/perception of self* *n* = 47 32.4%	*Self-esteem and negative self-talk*	Refers to children with low self-esteem evident in verbal statements and behavior	10 (66.7)	5 (33.3)	15 (31.9)
	*Depression symptoms*	Refers to symptoms of depression including suicidal ideation, generalized discouragement, and low energy	6 (42.9)	8 (57.1)	14 (29.8)
	*Anxiety*	Refers to reports of intense continued stress, and verbal reports of anxiety	5 (50)	5 (50)	10 (21.3)
	*Distress and suffering*	Verbal expressions of distress, unhappiness, and overall suffering	0 (0)	4 (100)	4 (8.5)
	*Disturbed body image*	Distorted perception of one's own body	0 (0)	1 (100)	1 (2.1)
	*Asking for help*	Refers to children asking to see a mental health care provider themselves	1 (100)	0 (0)	1 (2.1)
	*Attachment issues*	Refers to when a child becomes to attached or not attached enough to a caregiver for a period of time	1 (100)	0 (0)	1 (2.1)
	*Skewed perception of reality*	When child's report of an experience does not match other reports (for example from a teacher)	1 (100)	0 (0)	1 (2.1)
	**TOTAL**		**24** **(51.1)**	**23** **(48.9)**	**47** **(100)**
*Factors related to school* *n* = 34 23.4%	*Isolation*	Children withdraw from peers, self-isolating, loosing friends or refusing to participate in activities with others	5 (35.7)	9 (64.3)	14 (41.2)
	*Academic performance*	Children struggling academically or in need of increased support	3 (75)	1 (25)	4 (11.8)
	*Lack of motivation to attend*	Refers to a child’ lack of desire or aversion to attend school	5 (100)	0 (0)	4 (11.8)
	*Bullying and conflicts*	Instances of verbal or physical bullying or perpetual conflict with peers	2 (50)	1 (50)	3 (8.8)
	*Classroom disruptive behavior*	Unexpected or problematic behavior in the classroom setting	3 (100)	0 (0)	3 (8.8)
	*Non-adapted learning environment*	Refers to teachers untrained in special education and disability diagnoses and/or lack of mental health support in the classroom	3 (100)	0 (0)	3 (8.8)
	*Absences*	Repeated absences in school or unable to be present in class	0 (0)	2 (100)	2 (5.9)
	**TOTAL**		**21** **(61.8)**	**13** **(38.2)**	**34** **(100)**
*Behavioral manifestations* *n* = 22 15.2%	*Sleep related*	Factors related to difficulty sleeping.	4 (50)	4 (50)	8 (36.4)
	*Food related*	Factors related to behavior changes regarding to food refusal, selectivity, and appetite	1 (20)	4 (80)	5 (22.7)
	*Extreme behavior switches*	Children that become very reserved or start lashing out much more than previously	2 (66.7)	1 (33.3)	3 (13.6)
	*Communication patterns*	Changes in communication patterns with parents, HCPs, or peers	1 (50)	1 (50)	2 (9.1)
	*Affect*	Changes in emotional affect and interest in play	0 (0)	2 (100)	2 (9.1)
	*Extreme weight fluctuations*	Rapid changes in weight: weight gain or sudden weight loss	0 (0)	1 (100)	1 (4.5)
	*Hygiene*	Changes in hygiene or care for hygiene	0 (0)	1 (100)	1 (4.5)
	**TOTAL**		**8** **(26.3)**	**14** **(73.7)**	**22** **(100)**
*Physical manifestations* *n* = 18 12.4%	*Physical aggression*	Refers to physical reactions regarding aggression either verbal or physical	2 (33.3)	4 (66.7)	6 (33.3)
	*Agitation*	Increased physical movement	2 (50)	1 (50)	3 (16.7)
	*Headaches*	–	0 (0)	2 (100)	2 (11.1)
	*Self-harm*	–	0 (0)	2 (100)	2 (11.1)
	*Disorganization*	–	0 (0)	2 (100)	2 (11.1)
	*Picking scabs*	–	1 (100)	0 (0)	1 (5.6)
	*Increased spasticity*	Disordered sensorimotor muscle control	0 (0)	1 (100)	1 (5.6)
	*Fainting*	Fainting due to extreme emotions and/or anxiety	1 (100)	0 (0)	1 (5.6)
	**TOTAL**		**6** **(33.3)**	**12** **(66.7)**	**18** **(100)**
*Factors related to family dynamics* *n* = 8 5.5%	**–**	Refers to parental exhaustion, distress, stress or factors related to financial or familiar precarity	2 (25)	6 (75)	8 (100)
	**TOTAL**		**2** **(25)**	**6** **(75)**	**8** **(100)**
*Emotional dysregulation* *n* = 8 5.5%	**–**	Refers to lack of ability to adequately self-regulate emotions (crying, lashing out, lack of ability or vocabulary to express emotions)	1 (12.5)	7 (87.5)	8 (100)
	**TOTAL**		**1** **(12.5)**	**7** **(87.5)**	**8** **(100)**
*Mental health crises* *n* = 8 5.5%	*Psychosis*	Refers to a disconnection with external and physical reality	0 (0)	1 (100)	1 (12.5)
	*Panic attacks*	Intense episodes of anxiety accompanied with physical symptoms	0 (0)	1 (100)	1 (12.5)
	*State of crisis*	Repeated and uncontrolled tantrums and crises	2 (40)	3 (60)	5 (62.5)
	*Immediate threat of suicide*	Child threatening verbally or taking steps to commit suicide	1 (100)	0 (0)	1 (12.5)
	**TOTAL**		**3** **(37.5)**	**5** **(62.5)**	**8** **(100)**
**GRAND TOTAL**			**65** **(44.1)**	**80** **(55.9)**	**143** **(100)**

*CG04 (mother of two children with DCD—age 6, and ASD—age 8): “Then his teachers, the school psychologist […] they notice when he has these like physical or reactive behaviors. They tell me about them, and the response is generally like yeah, I know, he's already on the waiting list. Yeah, yeah, I know. We're already paying for private therapy. Like, yeah, I know. So, they're naming a problem, as in, they're naming how the problem manifests at school [but] they're not signaling that it exists”.*

#### 3.2.2 THEME: mental health care provided by HCPs

HCPs mentioned addressing pediatric mental health in numerous ways among the pediatric patients they see in their practice (Table 6). These included three main subthemes: direct, indirect, and group care. In direct care, most salient approaches were putting in place different forms of therapy and intervention plans to address the child's needs (*n* = 14 utterances, 35% of the subtheme), applying active listening (*n* = 11, 27.5%), and providing the child with emotional regulation tools (*n* = 7, 17.5%):

**Table 6:** Mental health care provided by HCPs for children with NDDs and their families.

Mental health care provided **by HCPs**			
Theme *n* = number of utterances % of Theme	Subtheme	Description	HCPs’ utterances *n* (%)
*Direct care* *n* = 43 43%	*Different therapy*	Forms of therapy and intervention plans to address the child's needs	14 (32.6)
	*Active listening*	Listening and paying intentional attention to the child's needs and words	11 (25.6)
	*Emotional regulations tools*	Provide the child with tools to improve their emotional self-regulation	7 (16.3)
	*Mental health preventative screening and evaluation*	–	4 (9.3)
	*Consult the child's environment*	Pay attention to the environment of the child, school and home life	3 (7.0)
	*Celebrate child's success*	Support the child in their progress	2 (4.6)
	*Involve child in care*	Include the child in the discussions and decisions regarding their mental health care	1 (2.3)
	*Less focus on diagnosis only*	Shift to overall wellbeing and less of a sole focus on the child's diagnosis	1 (2.3)
	**TOTAL**		**43** **(100)**
*Indirect/Referrals* *n* = 31 31%	*Referring out to other services*	Referring child to other more specialized care	25 (80.6)
	*Consulting with other HCPs*	Drawing on the knowledge from other HCPs in interdisciplinary teams	3 (9.7)
	*Advocating for child*	Help the child and family get the care they need in through the healthcare system	3 (9.7)
	**TOTAL**		**31** **(100)**
*Group care* *n* = 26 26%	*With the whole family*	Assess parental wellbeing, educate parents, involve and support the siblings	15 (57.7)
	*Group interventions*	Mentor groups for children, summer camps, parent support groups, self esteem conferences	11 (42.3)
	**TOTAL**		**26** **(100)**
**GRAND TOTAL**			**100** **(100)**

*HCP02 (SW): “Also talking directly with the student themselves, 1 on 1 with parent consent and just trying really more of a talk therapy approach. I sort of figure out what's going on with the perspective of the child is or youth and yeah. Some strategies from acceptance and commitment therapy or like- cognitive based type interventions. [But] I don't do CBT, but I draw from it”.*

Few HCPs reported using standardized mental health screening and evaluation (*n* = 4, 10%).

In the indirect care, clinicians reported to referring the child/family to a more specialized service (*n* = 25, 80.6%), consulting with other professionals and advocating for the child's needs (*n* = 3, 9.7% each). Overall, the tipping point to refer to more targeted services was at a low threshold. For some, a referral takes place once there was an official diagnosis or consultation with another clinician, however, most HCPs based this choice on personal clinical judgment:

*HCP03 (OT): “To be honest with you, because I'm not using anything really like standardized, or I don't have a questionnaire, sometimes it's just based on like my clinical judgment, but I do rely a lot on collaborating with the social worker to be like okay, I noticed this, this was said, or parents mentioned this […]. Where do we go from here?”*

Other factors that were found to precipitate a referral are in the order of complexity of the situation, “many risk factors”, or when the red flags laid out in Table 5 are very impactful for the child. Some HCPs reported attempting to mediate mental health issues that arise, others feel powerless, and for many, any mental health care is perceived to be outside of their mandate.

*HCP05 (OT): “I think it would have to be like, is it out of our mandate. You know, sometimes the mental health challenges might be in the context of having the physical impairment, sometimes it's goes beyond that, it might be more generalized anxiety, or you know, other types of things that are not necessarily only related to physical impairments. So that's where we would definitely refer out”.*

Group interventions (*n* = 19 utterances, 21.1% of the Theme) consisted of involving caregivers and siblings, as well as directing children to mentorship groups, summer camps, and relevant conferences/workshops (e.g., webinar on self-esteem).

#### 3.2.3 THEME: barriers and facilitators to pediatric mental health services

HCPs reported different barriers (Table 7A) and facilitators (Table 7B) they faced when addressing mental health in their clinical settings. The most important barriers that emerged are factors related to the healthcare systems (*n* = 82 utterances, 68.2% of theme). For instance, participants reported lack of targeted services, limited accessibility, and long waitlists:

**Table 7A:** Barriers to mental health care from the perspective of HCPs.

Barriers			
Theme *n* = number of utterances % of Theme	Subtheme	Description	HCPs’ utterances *n* (%)
*Factors related to the healthcare systems* *n* = 82 68.3%	*Existence of sufficient services, resources, and HCPs*	Lack of services geared towards mental health care in addition to lack of hired HCPs (notably psychologists), and resources unequally distributed across different administrative regions	31 (37.8)
	*Accessibility to services*	Inaccessible services either because of admission criteria, referral issues, non-adapted services for children with disabilities or location	16 (19.5)
	*Long waitlists*	Long waiting lists for medical appointments across the board leading to delays of care	13 (15.9)
	*Communication and collaboration between programs*	Refers to a lack of follow-up and communication post-referrals between siloed services	10 (12.2)
	*Focus on mental health*	Mental health care is not usually a main focus in rehabilitation care settings for example, or it is not addressed first when needed	8 (9.8)
	*Accessibility to information, or contacts*	Challenges related to accessing information, services or contact within the pediatric care sites (or lack of research in general)	4 (4.9)
	**TOTAL**		**82** **(100)**
*Factors related to caregivers* *n* = 14 11.7%	*Wellbeing*	CGs’ mental and physical state impacting their ability to support their children's mental health care	7 (50)
	*Uninvolved/disengaged*	Refers to CGs who are uninvolved and/or disengaged (during medical visits for example)	5 (35.7)
	*Previous experiences*	CGs with previous negative experiences with HCPs and/or the healthcare system (for example mistrust of social workers)	2 (14.3)
	**TOTAL**		**14** **(100)**
*Factors related to HCPs* *n* = 10 8.3%	*Lack of training and experience*	HCP not receiving enough training on mental health related care in general and for children with disabilities	7 (70)
	*Unclear interdisciplinary boundaries*	HCPs who work in interdisciplinary teams often find themselves uncertain as to whom should address certain issues	2 (20)
	*High personnel turnover*	Loss of personnel in addition to institutional knowledge	1 (10)
	**TOTAL**		**10** **(100)**
*Time limitation* *n* = 8 6.7%	–	Refers to time limitations during interactions (feeling of being rushed through medical visit or limited appointment time because of ongoing case load of the HCP)	8 (100)
	**TOTAL**		**8** **(100)**
*Mental health stigma* *n* = 3 2.5%	–	Refers to preconceived or stereotypical beliefs about mental health and it's treatment from CGs and/or HCPs	3 (100)
	**TOTAL**		**3** **(100)**
*Factors related to patients* *n* = 3 2.5%	–	Refers to various complications when it comes to providing care for the patients (medication challenges, overlapping diagnoses, overarching sociological barriers)	3 (100)
	**TOTAL**		**3** **(100)**
**GRAND TOTAL**			**120** **(100)**

**Table 7B:** Facilitators to the mental health care from the perspective of HCPs.

Facilitators			
Theme *n* = number of utterances % of Facilitators	Subtheme	Description	HCPs’ utterances *n* (%)
*Factors related to the healthcare systems* *n* = 28 53.8%	*Interdisciplinary team*	Having different professionals on the same team (psychoeducators, social workers, speech therapists…)	22 (78.6)
	*Collaboration and communication*	Effective inter-service and intra-service collaboration	3 (10.7)
	*Limited hours with patients*	Refers to the benefit of focusing on the most pressing issues through the service	1 (3.6)
	*Long term follow-up possibilities*	Refers to services that offer long term follow-ups for children and families	1 (3.6)
	*Support groups*	Refers to the services that provide spaces for children or parents to interact with peers going through similar experiences	1 (3.6)
	**TOTAL**		**28** **(100)**
*Factors related to HCPs* *n* = 11 21.2%	*Partnership with other HCPs*	Working closely alongside other clinicians with similar or different backgrounds outside	5 (45.5)
	*Knowledge of available services*	Knowledge of available services and referral services possibilities	3 (27.3)
	*Surveying patient's environment*	Refers to the HCP being willing and able to survey all of the different environments the patient is in (school, other services, siblings, etc.	2 (18.2)
	*Openly talking with the patients about diagnosis*	HCP who lacks awareness about certain cultural factors that can be at play and how to bridge them during interactions	1 (9.1)
	**TOTAL**		**11** **(100)**
*Factors related to caregivers* *n* = 9 17.3%	*Parent participation*	CGs who are involved and engaged in their child's health care services	6 (66.7)
	*Wellbeing*	CGs who are resilient and take care of their own mental health through family-centered care or therapy	3 (33.3)
	**TOTAL**		**9 (100)**
*Decreased mental health stigma* *n* = 2 3.8%	–	Refers decreased to preconceived or stereotypical beliefs post Covid-19 pandemic	2 (100)
	**TOTAL**		**2 (100)**
*Factors related to patients* *n* = 2 3.8%	*Communication and cognitive abilities*	Refers to children's ability to communicate with HCPs (about their distress, their emotions, their desires for example)	2 (100)
		**TOTAL**	**2** (**100)**
**GRAND TOTAL**			**52 (100)**

*HCP02 (SW): “So physical disabilities and like communication ones […] have less referral opportunities. Because if they require assistance, let's say with toileting or feeding, even hospitals have full-on, well, tried or refused in the past a client who needed physical care while they were in psychiatry. OK, let's be real. I've had clients who have been refused or offered limited intervention because of language. So, this [psychiatry] department doesn't speak English, so a student in severe distress [was sent out through] the revolving door because they couldn't provide an adapted inpatient experience in the hospital”.*

This finding is also reflected in the survey results, where CGs indicate that for the majority, their child is/was on a waiting list for mental health services, as well as in negative experiences, as the most commonly reported factor (Table 8, *n* = 10 utterances, 27% of negative experiences subtheme).

**Table 8:** Experiences of families of children with NDDs receiving mental health care.

Experiences of mental health care			
Theme *n* = number of utterances % of Theme	Subtheme	Description	CGs’ utterances *n* (%)
*Positive* *n* = 41 52.6%	*Specialized HCPs*	Neuropsychologist, OT, speech therapist, psychiatry, psychoeducator, school psychologist, social worker, special care counselor, special education technitican	23 (56.1)
	*Other*	HCPs advocating for families to get off the waitlist after they had fallen through the cracks, feeling supported when on the waitlist	6 (14.6)
	*Private therapy*	Private therapy and care accessed	5 (12.2)
	*Community support groups*	Community or parent support group for the parents and/or family	3 (7.3)
	*Family therapy*	Therapy geared towards the whole family	2 (4.9)
	*Hospitalization*	Care during a hospitalization	1 (2.4)
	*Fast assessment*	Rapid disability assessment mediated by the school	1 (2.4)
	**TOTAL**		**41** **(100)**
	*Long waitlists*	Children put on very long waitlists before receiving care	10 (27)
	*Other*	Had to access care through the private system, issues related to Covid, lost referral	9 (24.3)
*Negative* *n* = 37 47.4%	*“Unhelpful”*	Unhelpful experiences	7 (21.6)
	*Inaccessible services*	Services that are inaccessible for certain families or non-existent	6 (16.2)
	*Lack of personnel*	Not enough personnel for the need	2 (5.4)
	*Situations of conflict of interest*	Situations where the HCP did not disclose a conflict of interest with the CG	1 (2.7)
	*Incompetence*	Lack of competence from HCPs	1 (2.7)
	*Inappropriate comments*	HCPs berating CGs and their families	1 (2.7)
	**TOTAL**		**37** **(100)**
**GRAND TOTAL**			**78** **(100)**

The second main barrier mentioned includes factors related to CGs (*n* = 14, 11.7% of barrier-theme), including their level of engagement, well-being, and negative past experiences:

*HCP13 (Psychoeducator): “What is the hardest, in reality, is that we usually have parental distress […]. So that is where we try to offer support, but we are limited because it's an occupational or physical therapy service, so we don’t have any social work or psycho-educator. We are very limited in the services offered”.*

This finding is further reflected in the survey, where half of participating HCPs reported that CGs of children with NDDs would “*Often*” benefit from mental health services for themselves. In addition, CGs mentioned that a positive mental health care experiences encompassed family therapy, where interventions were geared towards the whole family and not only the child (Table 8, *n* = 2, 4.9% of positive experiences subtheme).

Facilitators ranged across similar themes to the barriers, as shown in Table 7B. The most mentioned enabler was the presence of an interdisciplinary team (*n* = 22 utterances, 78.6% within the factors related to healthcare systems) in which there is an HCP with expertise in mental health. Parallelly, CGs conveyed that most of their positive experiences were when specialized HCPs were engaged in mental health care (e.g., neuropsychologists, OTs, psychologists, Table 8, *n* = 23, 56.1% of positive experiences subtheme). Furthermore, HCPs reported that the CGs' engagement and well-being are important facilitators (*n* = 9, 17% within all facilitator utterances).

*HCP11 (PT): English translation: “The youth will go a lot farther if our parents are resilient, work through their grief, have less depressive symptoms, and are more available than those who have mental health problems”.[English translation]*

*HCP02 (SW): “What facilitates is definitely parent participation. So, if a parent is, you know, also either identifying a problem in the area of like a mental health problem, and they're talking about it and identifying it. That's always like 50% of the intervention”.*

*HCP16 (OT): “A big component in our team I'd say to really, you know, get the parents more involved in the rehab process. We like to use the term of getting them “de les embarquer” [“to unboard them”], you know to get them in the rehab process. And that's key because the more they see the value of our services, the more we can help them and that hopefully we're promoting you know a positive progression but that that is in and of itself, one of the obstacles in a way like in our practice”.*

In relation to this finding, the survey revealed that 90% of CGs are actively involved in their child's mental health management.

#### 3.2.4 THEME: positive and negative experiences of CGs within the pediatric health care systems

Reported positive and negative experiences are displayed in Table 8. On average, CGs conveyed having approximately one negative experience for every positive experience when receiving mental health evaluation and interventions for their child (*n* = 41 and *n* = 37 utterances, respectively). Negative mental health care experiences were largely attributed to the inaccessibility of services (*n* = 6 utterances, 16.2% within the negative experiences theme). The reasons mentioned spanned from long waitlists (*n* = 10, 27%) or referral problems (*n* = 2, 5.4%) to unhelpful or inadequate services once those are received (*n* = 8, 21.6%).

*CG07 (Father of three children with attention deficit hyperactivity disorder, age 10, language disorder, age 12, and DCD, age 12): “It's not accurate to say we've not received any services, but it's accurate to say we've not received any meaningful services”.*

Some less common yet important occurrences were CGs' experiences related to HCPs lacking competence (*n* = 1 utterance, 2.7% of negative experiences theme), a blatant conflict of interest (*n* = 1, 2.7%), or receiving inappropriate comments from HCPs (*n* = 1, 2.7%):

*CG05 (Mother of a child with Duchenne muscular dystrophy, age 20): “[She] made me feel like I was the worst parent on this planet, […] she at one point she was like, she wanted to place him, like there was something wrong with me […] but you're made to feel like it's your fault, like […] maybe you're too permissive, or maybe you're too this, or maybe you're too that, or you know like the way your parent is bad”.*

This subtheme is complemented by the survey finding, where 50% of CGs felt being only “*Sometimes*” supported by the HCPs from whom they received care for their child. On the other hand, positive experiences were reported once care was from a specialized HCP (e.g., psychologist, OT, *n* = 23 utterances, 56.1% of positive experiences subtheme). However, some of the positive services were accessed through the private system (*n* = 5, 12.2%).

Impacts of mental health services are displayed in Table 9. Once families have successfully received services, CGs mainly noted “positive” impacts (*n* = 15 utterance, 83.3% of impacts theme) on their children with NDDs, and less often, “none” or “harmful” impacts (*n* = 3, 16.7%):

**Table 9:** Impact of the mental health care received.

Impact of care			
Theme *n* = number of utterances % of tHEME	Subtheme	Description	CGs’ utterances *n* (%)
***Positive*** *n* = 15 83.3%	*Attention from HCPs*	Benefits from having a professional spending time and talking with the child	6 (40)
	*Impact on whole family*	Positive impact on the whole family dynamics at home	3 (20)
	*Better daily functioning*	Child functions better on the day to day- at school and at home	3 (20)
	*Expressing feelings*	Children able to better express their emotions and how they feel in general or regarding their disability	2 (13.3)
	*Information on disabilities*	Gained helpful information regarding a diagnosis	1 (6.7)
	**TOTAL**		**15** **(100)**
***Negative*** *n* = 2 11.1%	*Harmful*	The care inflicted more harm on the child and the family	1 (50)
	*No immediate effects*	The care results are not seen directly	1 (50)
	**TOTAL**		**2** **(100)**
***Neutral*** *n* = 1 5.6%	*–*	Care has been “more or less” helpful	1 (100)
	**TOTAL**		**1** **(100)**
**GRAND TOTAL**			**18** **(100)**

*CG10 (Father of a 6-year-old boy with ASD): “ The impact was [related to improvements] in my son's daily life. He was talking, [had] fewer breakdowns, so that was the best improvement—fewer breakdowns in his daily life”. Translated from French*.

*CG04 (Mother of an 8-year-old boy with DCD and a 6-year-old boy with ASD): “My 8-year-old is more articulate about what is happening internally for him and will ask me for moments to talk, and he will name his emotions. My 6-year-old will name what is happening in his body or in his mind in his experiences. He's very articulate about what it feels like to be in his brain or in his body at any moment. And again, that's, I'm at this point lumping in OT and speech as self-awareness as things that are super necessary for mental health in neurodivergent people”.*

*CG06 (Mother of an 8-year-old boy with DCD): “He sees school in a positive way, he sees friends in a positive way, and then he thinks about other people besides himself as well there”.*

#### 3.2.5 THEME: solutions to improve mental health services

A broad range of improvements (eight subthemes) to the current systems were recommended (Table 10). The most commonly reported solution related to services' accessibility (*n* = 50 utterances, 25.8% within Theme), which included recommendations to shorten the waitlist (*n* = 16, 32% within the accessibility theme), broaden inclusion criteria with less focus on diagnosis and more focus on actual needs (*n* = 10, 20%), as well as to rectify inequalities (e.g., due to financial situation, location, and language) (*n* = 7, 14%).

**Table 10:** Solutions proposed to improve mental health care for children with disabilities from the perspective of HCPs and CGs.

Improvements					
Theme *n* = number of utterances % of Theme	Subtheme	Description	CG's utterances *n* (%)	HCPs’ utterances *n* (%)	Total utterances *n* (%)
Service accessibility *n* = 50 25.8%	*Shorten waitlists*	Refers to less delays between time a service is request and receiving care/services while on the waitlists	8 (50)	8 (50)	16 (32)
	*Change exclusion criteria, less focus on diagnosis*	Provide services based on needs rather than diagnostic criteria or age, for example	1 (10)	9 (90)	10 (20)
	*More available services*	More useful services provided for children and families	6 (60)	4 (40)	10 (20)
	*Adapted care for services/therapies*	Provide adapted care to children who need extra care: such as personal care support, adapted communication tools for example	3 (50)	4 (50)	6 (12)
	*Financial accessibility (private inaccessible)*	Access to services in the public system for families that can’t afford to access private care	4 (100)	0 (0)	4 (8)
	*Language options*	Provide services in the language needed (for example more Anglophone services)	2 (100)	0 (0)	2 (4)
	*Different accessibility modalities*	Refers to provides services available in beneficial times and manners: for example on weekends, virtually, or through texting	0 (0)	1 (100)	1 (2)
	*Location disparity*	Inequalities based on administrative region	1 (100)	0 (0)	1 (2)
	**TOTAL**		**25** **(50.0)**	**25** **(50.0)**	**50** **(100)**
*Rethinking services* *n* = 36 18.6%	*Proactive approach to MH*	Refers to mental health care being viewed as a preventative and proactive step in all areas of care rather than when the need becomes dire	2 (14.3)	12 (85.7)	14 (38.9)
	* Decouple MH and disability*	Decouple children with disabilities’ mental health problems from their disability itself: access to needed services outside of disability programs	3 (60)	2 (40)	5 (13.9)
	*MH screening tool*	Establish a mental health screening tool that can be used to identify and prevent mental health issues	0 (0)	4 (100)	4 (11.1)
	*Provide needed services*	Provide services that are actually needed (therapy and assessments for example)	4 (100)	0 (0)	4 (11.1)
	*Public-Private systems*	Improve public systems or hybridize systems	2 (100)	0 (0)	2 (5.6)
	*Integrate MH care*	Refers to integrating mental health care with disability programs	2 (100)	0 (0)	2 (5.6)
	*Changes in approach*	Focus on children: listening to their concerns, allowing them to be absent during parent reporting meetings	2 (100)	0 (0)	2 (5.6)
	*ABA therapy*	Caution in using ABA therapy to address mental health concerns	2 (100)	0 (0)	2 (5.6)
	*Focus on health as a whole*	Holistic approach to care: focus on health and not the absence of disease	0 (0)	1 (100)	1 (2.8)
	**TOTAL**		**17** **(47.2)**	**19** **(52.8)**	**36** **(100)**
*HCPs training and hiring* *n* = 34 17.5%	*Increased training for all HCPs*	Increased mental health care and disability training for HCPs of all backgrounds	4 (25)	12 (75)	16 (47.1)
	*More psychologists*	–	1 (14.3)	6 (85.7)	7 (20.6)
	*More specialized HCPs*	In addition to the above categories, hiring more mental health specialized HCPs (such as psychoeducators, and neuropsychologists for example)	2 (33.3)	4 (66.7)	6 (17.7)
	*More social workers*	–	2 (50)	2 (50)	4 (11.8)
	*Less HCP turnover*	Reduce the high HCP turnover in schools and programs for continuity of care and services	1 (100)	0 (0)	1 (2.9)
	**TOTAL**		**10** **(29.4)**	**24** **(70.6)**	**34** **(100)**
*Widening MH care target population* *n* = 31 16%	*Family-centered care*	Refers to care centered around the family (parents and siblings in addition to the child)	7 (36.8)	12 (63.2)	19 (61.3)
	*Focus on adolescence and transitional times*	Services adapted for this age group and during transitional life periods	4 (66.7)	2 (33.3)	6 (19.4)
	*Target young children*	Services adapted for younger children also experiencing mental health challenges	1 (33.3)	2 (66.7)	3 (9.7)
	*Caregiver specific support*	Services to help the CGs navigate the medical system itself	2 (100)	0 (0)	2 (6.5)
	*Children with complex communication needs*	Services adapted for the population of children with communication needs	0 (0)	1 (100)	1 (3.2)
	**TOTAL**		**14** **(45.2)**	**17** **(54.8)**	**31** **(100)**
*Service collaboration and communication* *n* = 22 11.3%	*Better inter-service links and communication systems*	Refers to instituting channels of communication, more collaboration internally and between different programs involved with the child	0 (0)	19 (100)	19 (86.4)
	*Standardization of care*	Standardization of services offered across locations, mental healthcare benchmarks, and standard questionnaires	0 (0)	3 (100)	3 (13.6)
	**TOTAL**		**0** **(0)**	**22** **(100)**	**22** **(100)**
*External policy shifts* *n* = 11 5.7%	*MH support in school setting*	Increased trained support at school- mental health practitioners	7 (77.8)	2 (22.2)	9 (81.8)
	*Policy to free caregivers from work*	Policy changes to improve availability and flexibility for parents and caregivers of children with disabilities	0 (0)	1 (100)	1 (9.1)
	*Disability laws*	Recognition of disability in Quebec needs to be broadened	1 (100)	0 (0)	1 (9.1)
	**TOTAL**		**8** **(72.7)**	**3** **(27.3)**	**11** **(100)**
*Better knowledge and description of services* *n* = 6 3.1%	**–**	Centralized description of services currently available and accessible to HCPs and caregivers	3 (50.0)	3 (50.0)	6 (100)
	**TOTAL**		**3** **(50.0)**	**3** **(50.0)**	**6** **(100)**
*Mental health stigma* *n* = 4 2.1%	–	Keep reducing mental health stigma post Covid-19 pandemic and including it more as a focus in health care services across the board	0 (0)	4 (100)	4 (100)
	**TOTAL**		**0** **(0)**	**4** **(100)**	**4** **(100)**
**GRAND TOTAL**			**77** **(39.7)**	**117** **(60.3)**	**194** **(100)**

*CG05 (Mother of a child with Duchenne muscular dystrophy, age 20): “[I’d like to see] that there's a space for someone with a physical disability to go. […] There's basically nowhere where anyone with physical needs that has mental problems can go. There's nowhere at all. And that's as an adult and as a child, it's just as bad. So yeah, somewhere with people with physical needs that they can go would be great, or at least […] offer the option [for adapted care] if you're willing to pay for [it]”*.

*CG01 (Mother of a child with ASD and ADHD age 12): “So you have to care about health in general, like mental health is important regardless of the language you speak, and so if in this area they need to hire more anglophones, well they should do that- like they need to do”.*

*HCP02 (SW): “Since we're talking about disability, for […] disabilities that require personal care support should really be prioritized because the minute we start excluding people from receiving services because personal care can't be provided within the setting or [otherwise] it just falls on caregivers”.*

Furthermore, the target population of services was recommended to be widened with an emphasis on family-centred care (*n* = 19, 61.3% within the widening mental health care population subtheme) and adolescents, as their mental health is particularly vulnerable during this transitional life stage (*n* = 6, 19.4%).

*HCP02 (SW): “I feel like when in adolescence, when we see an uptake, maybe in sort of mood changes even in our non-verbal students and youth, children, or youth. At that point, you know, we have to remember that the caregiver is also in a period of transition and aging, and so it's almost like the harder sometimes things can be. So, my hypothesis is that like something like, let's say it could be attention problems, it could be anxiety type symptoms that become more pronounced during adolescence”.*

*CG06 (Mother of a child with DCD, age 8): “I think we should pay attention to starting school, starting high school, these are difficult times for a child that does not have other problems. [For] a child that already has a diagnosis or a handicap, it just adds or elevates the difficulty level. [English translation]”*

When it comes to current systems in place, other improvements discussed were increased HCP's training and hiring (*n* = 34 utterances, 17.5% within Theme), better knowledge of services currently available (*n* = 6, 3.1%), and external policy shifts (*n* = 11, 5.7%). Finally, CGs and HCPs brought up themes of rethinking and reorganizing how services are administered and conceptualized (*n* = 36 utterances, 18.6% of Theme). Most notably, decoupling mental health issues from disability and adopting a proactive attitude towards mental health care rather than “*putting out fires*” (HCP01, Dietician):

*HCP01 (Dietician): “This should be on our radar all the time as clinicians […], we’re weighing patients, we’re like taking their blood pressure, for example. This is the kind of routine care that we do, and I feel like the only question we really asked them is how are you? This is something that would address mental health […]. Should this be on our list of to-do things with patients?”*

*HCP16 (OT)—“I think there's more and more research pointing towards the fact that people with disabilities are more at risk for mental health issues. So why do we not have more as a proactive lens?”*

## 4 Discussion

The objectives of this study were to explore CGs' and HCPs' perspectives and experiences related to mental health issues and mental health services for children with NDDs and their families. Additionally, we sought to describe common precursors to mental health challenges in this population. This comprehensive study sheds light on the complex landscape of mental health services for children with NDDs and their families, emphasizing the need for systemic improvements and a holistic, proactive approach involving both, the CGs and HCPs. Several intersecting findings between the quantitative (survey) and qualitative (interviews) have emerged and underscore the multifaceted challenges faced by this vulnerable population. The convergence of the data highlights the intricate dynamics surrounding mental health services. As we navigate the multifaced field, it becomes evident that collaborative efforts between caregivers and healthcare providers are imperative for crafting comprehensive solutions and fostering positive outcomes in pediatric mental health care.

A clear perceived need for mental health services for children with NDDs and their families from the perspectives of both study groups was noted. However, the study highlighted important challenges pertaining to accessibility and availability of targeted and comprehensive services. The results of this study further align with our environmental scan (25), as the lack of accessible care and the need to adapt care for children with NDDs have emerged. Furthermore, our results are congruent with a recent study that evidenced gaps in access and quality of mental health services for youth with NDDs (20). We determined that once services were accessed by families, many expressed being satisfied with them in terms of HCPs' understanding of the child's mental health needs. Nonetheless, our caregiver participants reported being only sometimes supported by HCPs, indicating room for improvement in caregiver support and empowerment. The well-being and engagement of caregivers of children with NDDs are paramount to the well-being of their children and the effectiveness of the care provided (37–39). Resilient and supported CGs can, in turn, effectively support their child (37). Our findings indicate that CGs were actively engaged in their child's mental health management. However, they also reported lacking resources and services for their mental health. Participating clinicians reported that a critical barrier to effective mental health care is limited caregivers' resilience in the face of their child's growing and changing needs, which could be affected by their own deteriorating mental health. This finding is consistent with previous reports on the need for mental health support and services for caregivers of children with disabilities (40, 41). Overall, our study further underscores the need for mental health services to integrate and embed a family-centered approach (42), where the child's and CG's mental health can be equally addressed.

In terms of referral practices, we found that HCPs often relied on personal clinical judgment rather than standardized tools, highlighting the need for more standardized approaches.

Moreover, we noted that clinicians expressed a modest sense of readiness to handle challenges in pediatric mental health, with a notable 87.5% indicating a lack of sufficient information regarding referral options. In fact, a frequently suggested solution in our study involved enhancing the training of HCPs. Boosting their exposure to this population through expanded clinical experience, active participation in professional development, engagement in knowledge translation initiatives, and fostering collaborations between clinical and academic realms could effectively promote the incorporation of evidence-based mental health practices into clinical settings (43, 44). Particularly, Scratch et al. (21), recommends a “formal upskilling for rehabilitation team members” (p. 365), where HCPs can learn about solution-focused strategies, suicide and risk assessment, other therapies (e.g., cognitive-behavioral, psychosocial), and form clear roles and responsibilities within their treating teams (21). In fact, staff training has been previously highlighted as one of the top three key principles in pediatric mental health care, along side family involvement and accessible care (20). Interestingly, this suggestion is echoed not only by participating HCPs in our study, but also by CGs.

Furthermore, the complexity of the situation and impactful precursors to mental health challenges were noted as factors influencing HCPs decision to refer. As such, our study identified several red flags/precursors to mental health challenges. These factors, signs, and symptoms, could be used to recognize potential issues early, promote a preventative approach, pinpoint, and inform the need for care, and serve as a conversation icebreaker and facilitator in CGs—HCPs discussions about pediatric mental health. Moreover, HCPs reported that caregivers’ identifying a mental health problem in their child constitutes a facilitator. Therefore, raising awareness about common precursors to mental health issues in children with NDDs is a warranted avenue.

The emerging barriers to pediatric mental health care, including factors related to healthcare systems, inadequate communication and collaboration among healthcare professionals, and a lack of coordination and continuity of care, have also been reported in previous research conducted in similar and different settings. For instance, Majnemer et al. identified a lack of educational and rehabilitation resource support and psychology services for children with CP in school settings (45). Excluding individuals with NDDs from research trials and conversations around educational reform can also contribute to the lack of collaboration and communication between the system and this population (46). These limitations hinder the accessibility and effectiveness of mental health services for children with NDDs and their families, and further research should evaluate the implementation of possible solutions identified. To render mental health services more accessible, waitlists can be targeted. For instance, implementing services while waiting is one possible solution [e.g., caregiver coaching, online telehealth services (47, 48)]. Resources distribution in addition to the clinical capacity to work with this vulnerable population (notably psychologists and social workers) or alternative delivery of services through telehealth are additional suggestions (26, 27). Telerehabilitation for children with NDDs and their families has shown to be more effective or as effective as traditional in-person approaches in improving various child- and parent-related outcomes (27). The field of telerehabilitation is evolving, and several low-cost, user-friendly, and evidence-based resources (ranging from informational articles and one-on-one health coaching with a professional) are currently available to clinicians and families to facilitate providing and receiving care using this model (49).

Furthermore, access to publicly available therapy for families, especially those with a lower socioeconomic status, is paramount in the rise of the privatization of care (50). Mental health programs for youth with NDDs and their families outside of the rehabilitation centers present with restricting options. This might be due to factors such as unadapted telemedicine options (51) or organizational barriers (e.g., lack of funding, high employee turnover) (52). CGs in our study advocated for decoupling mental health issues and the diagnosis. This would entail the removal of exclusion criteria parallel to instituting the infrastructure for personal care personnel in non-rehabilitation settings. To support children with NDDs, mental health services must cater to a broader population and be made publicly available. Moreover, children are more vulnerable during transitional periods (e.g., transition from daycare to a primary school environment, adolescent transition) (53). Therefore, increased services should be available to address these periods in the healthcare settings and within the school systems. The school setting was identified as a beneficial target, corroborating previous findings (54). However, barriers exist, such as lack of personnel, funding, and time constraints. Increased initiatives about mental health awareness (e.g., lunchtime workshops) and therapy for youth with NDDs could be provided by specialized personnel in school settings (55). This would address the critical needs and parallelly ease the burden on CGs.

Lastly, mental health care must be considered an integral and essential part of medical services for children with NDDs (3, 54). This requires shifting to a more proactive rather than reactive system and establishing a standard of mental health care across locations and professions. These suggestions are also reflected in the recently proposed treatment-oriented biopsychosocial framework for care (21). Furthermore, instituting standardized tools, mental health care training for HCPs, and more rapidly accessible services will require altering priorities and redistributing resources. Though increasingly destigmatized, mental health needs to be included in frontline treatment. Our recommendations are inline with the Canadian Mental Health Strategy (56), where mental health issues are recommended to be treated on the same level and of the same manner than other chronic health conditions, promote multidisciplinary mental health management, and upskilling HCPs such that they develop and maintain evidence-based mental health practices.

## 5 Limitations

The chosen sampling method has the disadvantage of potentially introducing a selection bias, as participating HCPs could be more invested in their patients mental health, and the CGs could have more negative experiences. Though the sample size was small, the diversity in characteristics of CGs, their children, and HCPs enhanced the results generalizability beyond the study context. Furthermore, participants from a limited geographical area were included, which may not represent a diverse population. The questionnaires used in this study were designed in-house, which could affect the validity of the findings. Nonetheless, clinical and patient-partners were engaged in the co-design process to ensure that the questionnaires incorporated diverse perspectives and relevant insights, enhancing the robustness of the study's methodology.

## 6 Future directions

Considering the results of this study, it is evident that there is a continued need for improvements in pediatric mental health management. Present findings can inform future knowledge mobilization and implementation science research. Our team is currently working on developing a knowledge translation toolkit to increase healthcare professionals' and caregivers' awareness about mental health triggers in children and youth with NDDs. Moreover, we developed a search database of local in-person [In-person Programs—Resi-Alliant Kid Lab (resialliantkidlab.com)] and online mental health services [Online Programs—Resi-Alliant Kid Lab (resialliantkidlab.com)]. This database is open-access, where a user can search a service based on commonly employed keywords (e.g., depression, anxiety, autism). The search generates a list of available services, the requirements/admission criteria of the program, the target of the program, its mandates (e.g., assessment vs. treatment), and contact information. We highlight the need for increasing the access and priority of publicly available mental health services for children, specifically with NDDs. To that effect, we propose to build a community of key stakeholders, such as patient partners (including caregivers of children with NDDs and youth/young adults with NDDs), clinicians, researchers, policymakers, and community representatives, creating partnerships to develop and implement initiatives to continue advocating for the optimization of the field of pediatric mental health.

## 7 Conclusions

In conclusion, our findings underscore the need for a more systematic and standardized approach to mental health services for children with NDDs and their families. Improving caregiver support and addressing barriers, especially system-related issues, are critical for enhancing mental healthcare accessibility and quality. The proposed solutions, including a proactive attitude and a focus on family-centered care, offer valuable insights for shaping policies and practices in pediatric mental health services.

## Data Availability

The raw data supporting the conclusions of this article will be made available by the authors, without undue reservation.
