# Supplementary material for: Mental health in children with disabilities and their families: red flags, services' impact, facilitators, barriers, and proposed solutions
Source: Front Rehabil Sci. 2024 Feb 12;5:1347412. doi: 10.3389/fresc.2024.1347412 (PMC10894921; doi:10.3389/fresc.2024.1347412)
Supplement: Supplementary file 1 [file Datasheet1.pdf]

Supplementary File 1

Study Materials

<u>For caregivers:</u>

**Child/youth**<br>*More than one child with a disability, specify each and differences	
**Age of child (years)**	years old
**Race/ethnicity of child**	☐Caucasian ☐Black/African ☐Asian ☐Native ☐Hispanic/Latino<br>☐Other:
**Gender of child**	☐ Female ☐ Male ☐ Prefer not to disclose
**Does your child have a disability?**	☐ Yes ☐ No
**If yes….**	
What is the primary disability that causes difficulty for your child?	
Does your child have any secondary disabilities?	
**Does your child have other health conditions that are important to note?**	
**Number of siblings?** Leave blank if none	
**Rank of Child** (order of birth)	

**Caregiver**	
**Age**	years old
**Gender**	
**Race/Ethnicity**	☐Caucasian ☐Black/African ☐Asian ☐Native ☐Hispanic/Latino<br>☐Other:
**Relation to the child**	☐ Biological ☐Foster ☐Step ☐legal guardian Other:
**Marital Status**<br>*More than one option might apply	☐ Married ☐Single ☐Divorced ☐Blended family (e.g. remarried)<br>☐Widowed ☐Same-sex couple ☐Prefer to not state
**Education Level**	
**Employment: occupation & status**	**Occupation:**<br>☐Full-time ☐Part-time ☐Unemployed ☐Retired ☐On disability ☐On leave

Supplementary File 1

	Not at all-Never	Slightly-Sometimes	Moderately-Often	Very much-All the time	N/A
**The following statements are about awareness and accessibility of mental health-care services for your child with a disability.**					
Over the past year, mental health services for my child have been easily accessible (e.g., location, availability, cost, admission criteria).	☐	☐	☐	☐	☐
Over the past year, mental health care services were declined for my child because they did not meet the admission criteria.	☐	☐	☐	☐	☐
Over the past year, mental health care services were declined for my child because of the location.	☐	☐	☐	☐	☐
Over the past year, I have had to refuse mental health care services because of associated costs (e.g., consulting in a private clinic).	☐	☐	☐	☐	☐
Presently, or in the past, my child has been on a waiting list for mental health services.	☐	☐	☐	☐	☐
Do you feel that you were adequately informed and aware of the mental health services offered for your child	☐	☐	☐	☐	☐
I had positive/good experiences/helpful interactions with health care services related to my child's mental health.	☐	☐	☐	☐	☐
I had negative/bad experiences/not helpful interactions with health care services related to my child's mental health.	☐	☐	☐	☐	☐

**The following statements are about your satisfaction with the mental health services offered by HCPs over the last year.**					
	Not at all-Never	Slightly-Sometimes	Moderately-Often	Very much-All the time	N/A
Relationship with HCPs:					
I feel my child's mental health has been generally supported by HCPs.	☐	☐	☐	☐	☐
As a caregiver, I feel generally supported by HCPs in relation to my child's mental health.	☐	☐	☐	☐	☐
Overall, I am satisfied with the mental health services my child has received.	☐	☐	☐	☐	☐
Overall, I am satisfied with the interactions/communication I had with HCPs in the past year about my child's mental health.	☐	☐	☐	☐	☐
I have felt HCPs spent time understanding the situation and addressing my child's mental health needs.	☐	☐	☐	☐	☐
Mental health management provided by HCPs in relation to my child's mental health *(e.g., evaluation, referral, follow-up, treatment, consultation):*					
I have felt informed about my child's condition during my child's mental health management support.	☐	☐	☐	☐	☐
I have been actively engaged in my child's mental health management (e.g., being present at evaluation, involved in the choice of treatment options, applying intervention strategies with my child).	☐	☐	☐	☐	☐
The mental health services received have had a positive impact on my child.	☐	☐	☐	☐	☐
The mental health services received have had a positive impact on me as a caregiver.	☐	☐	☐	☐	☐

Supplementary File 1

Semi-Structured interview questions for CGs

During the audio-recorded interview, the interviewer will be asking you the following questions. This is just a guide for the interviewer, and additional questions might be asked for clarification. You can take time before the interview to think about your answers. Text boxes are provided as an option to draft your responses. This semi-structured interview will take 30-45 minutes.

1) **Can you describe the red flags or precursors to the mental health concerns for your child?**
   a. Did your child show key signs of mental health problems which led you to seek mental health support? What were these key signs?
   b. Were there any signs identified by HCPs that initiated treatment and/or mental health support for your child?
   c. Did any other significant people in your child's life identify the need for mental health support (e.g., teachers, coaches, friends)?

2) **Can you describe, in general terms, your experience with mental health services for your child:**
   a. What types of services have you received?
   b. What types of services have been helpful (positive experiences) and why?
   c. What types of services have been less helpful or not helpful at all (negative experiences) and why?

3) **What impacts of the mental health services have you observed** (in your child and/or yourself as a caregiver, family functioning, etc.)?

4) **Based on these experiences, what do would you like to see improved regarding the mental health care for children with disabilities?**

Supplementary File 1

<u>For HCPs</u>

**Age (years)**	
**Gender**	☐Male ☐Female ☐Prefer not to disclose
**Occupation and status**	☐Full-time ☐Part-time ☐Currently unemployed ☐On leave
**Highest degree obtained & year of graduation**	
**Work description**	**Population served (e.g., children with cerebral palsy):**<br>**Setting (e.g., outpatient, inpatient)**:<br>**Experience in childhood disability (yy/mm):**

	**Not at all-Never**	**Slightly-Sometimes**	**Moderately-Often**	**Very much-All the time**	**N/A**
**Over the past year, I have had pediatric patients with the following condition(s) who would benefit from mental health services** (evaluation, treatment, follow-up):					
…Cerebral palsy	☐	☐	☐	☐	☐
…Autism spectrum disorder	☐	☐	☐	☐	☐
…Attention-deficit and hyperactivity disorder	☐	☐	☐	☐	☐
…Developmental coordination disorder	☐	☐	☐	☐	☐
…Learning disability	☐	☐	☐	☐	☐
…Intellectual disability	☐	☐	☐	☐	☐
…Global developmental delay	☐	☐	☐	☐	☐
…Speech disability	☐	☐	☐	☐	☐
…Other (please specify): ___________________________	☐	☐	☐	☐	☐

Supplementary File 1

The following statements are about your experience regarding mental health care management.					
Over the past year, I have had parents/caregivers of pediatric patients who would benefit from mental health services as part of family-centered care.	☐	☐	☐	☐	☐
Over the past year, I have had to refer patients and/or their caregivers to more targeted mental health program(s).	☐	☐	☐	☐	☐
I am adequately prepared to conduct pediatric mental health assessments.	☐	☐	☐	☐	☐
I am adequately prepared to treat pediatric mental health challenges.	☐	☐	☐	☐	☐
I am adequately prepared to manage parental mental health issues (e.g., parents' stress, depression, anxiety, mental breakdown, etc.).	☐	☐	☐	☐	☐
I feel well informed about referral possibilities for targeted mental health issues (e.g., acute crises, group therapy).	☐	☐	☐	☐	☐
I feel supported by my management in addressing mental health challenges in my pediatric clientele.	☐	☐	☐	☐	☐
I feel well informed about existing mental health care services/resources that are offered within my clinical setting and partnering sites.	☐	☐	☐	☐	☐
I feel well informed about existing mental health care services/resources that are offered outside of my clinical setting and partnering sites (e.g., another jurisdiction, online services).	☐	☐	☐	☐	☐

Semi-Structured interview questions for HCPs

During the audio-recorded interview, the interviewer will be asking you the following questions. This is just a guide for the interviewer, and additional questions might be asked for clarification. You can take time before the interview to think about your answers. Text boxes are provided as an option to draft your responses prior to the interview.

1) Describe how you address the mental health of children/youth with and without a disability in your practice.

2) Describe your experiences of interacting with families and providing mental health support for children/youth with and without a disability:

a. What facilitates your practice and why?
b. What are the obstacles in your practice, and why?

3) What are the key signs of mental health challenges in children/youth that you see (i.e., red flags)?

a. Is there a tipping point where you decide to refer to a more targeted program, and if yes, what is it?

4) Describe the impacts of the mental health services you provide to your pediatric clientele/their caregivers.

5) How can we improve mental health services for children with and without a disability?
